# A language-matching model to improve equity and efficiency of COVID-19 contact tracing

**DOI:** 10.1073/pnas.2109443118

**Published:** 2021-10-22

**Authors:** Lisa Lu, Benjamin Anderson, Raymond Ha, Alexis D’Agostino, Sarah L. Rudman, Derek Ouyang, Daniel E. Ho

**Affiliations:** ^a^Regulation, Evaluation, and Governance Laboratory, Stanford University, Stanford, CA 94305;; ^b^County of Santa Clara Public Health Department, San Jose, CA 95126

**Keywords:** language access, contact tracing, COVID-19, health equity, machine learning

## Abstract

Contact tracing is a pillar of COVID-19 response, but language access and equity have posed major obstacles. COVID-19 has disproportionately affected minority communities with many non–English-speaking members. Language discordance can increase processing times and hamper the trust building necessary for effective contact tracing. We demonstrate how matching predicted patient language with contact tracer language can enhance contact tracing. First, we show how to use machine learning to combine information from sparse laboratory reports with richer census data to predict the language of an incoming case. Second, we embed this method in the highly demanding environment of actual contact tracing with high volumes of cases in Santa Clara County, CA. Third, we evaluate this language-matching intervention in a randomized controlled trial. We show that this low-touch intervention results in 1) significant time savings, shortening the time from opening of cases to completion of the initial interview by nearly 14 h and increasing same-day completion by 12%, and 2) improved engagement, reducing the refusal to interview by 4%. These findings have important implications for reducing social disparities in COVID-19; improving equity in healthcare access; and, more broadly, leveling language differences in public services.

## Significance

Language discordance has been shown to contribute to social disparities in healthcare. Contact tracing is essential to combating COVID-19, but language differences between contact tracers and patients have hindered its efficacy. We demonstrate a general method for leveraging machine learning and administrative data to maximize the impact of bilingual contact tracers and level language differences. We evaluate in a randomized controlled trial the impact of language matching on high-volume contact tracing in Santa Clara County, CA, and show that it reduces time spent and improves engagement with contact tracers. These results illustrate the advantages of utilizing bilingual personnel over third-party interpreters in improving social services.

Contact tracing—the process of calling diagnosed cases, identifying and notifying their contacts, and assisting with isolation and quarantine—is a pillar of infectious disease response. Throughout the COVID-19 pandemic, many jurisdictions have rapidly scaled up contact-tracing efforts to contain the spread of the disease. Given the disproportionate impact COVID-19 has had on immigrant and minority communities, it is vital to ensure that contact tracing works effectively and equitably across different segments of the population. Language and cultural barriers may contribute to disease disparities if the same quality of care and contact tracing cannot be delivered to vulnerable communities. In this paper, we illustrate how machine learning can be used to level these language differences through a randomized controlled trial conducted with the County of Santa Clara Public Health Department’s Case Investigation and Contact Tracing (CICT) Team.

COVID-19 has highlighted the structural, language, and cultural barriers Latinx communities face when trying to comply with policies such as shelter-in-place orders. The disparity in impact is stark—38.9% of California’s population is Latinx, yet Latinx individuals make up 55.6% of COVID-19 cases and 46.6% of COVID-19 deaths (1, 2). A variety of factors are thought to have contributed to this acute disparity, from Latinx individuals being more likely to be employed in essential industries and therefore facing greater occupational exposure to COVID-19 (3–5) to the lower likelihood of having health insurance, making it difficult to access necessary testing and treatment resources (6). Combined with higher-density living conditions, mistrust of public health authorities, and insufficient information and resources in their preferred language, these factors put the Latinx community at heightened risk of poor health outcomes and COVID-19 (3–5, 7, 8). The outsized risk these communities face makes it all the more critical that contact tracers are able to reach these communities, interrupt new chains of infections, and provide supportive resources during isolation and quarantine.

Trust and rapport are critical for effective contact tracing (4, 9–11), as interviews must cover sensitive and private topics to identify contacts. These calls are also important for informing policymaking and case investigation, as the initial case reports from laboratories are sparse in information. However, patients in minority communities may be especially fearful of phone calls from the government, unwilling to share personal information about themselves or their networks (11), or worried that disclosing their information could affect their employment or immigration status (4). Contact tracers with local language fluency and cultural competency can build greater trust among minority groups and immigrant communities, engage with patients in their preferred language, and dispel myths and misinformation (9). These language differences can be addressed by providing contact-tracing interviews in a patient’s preferred language.

Santa Clara County, CA, home to the city of San Jose, is a county with 1.9 million residents. It was the first US jurisdiction to issue a shelter-in-place order, in coordination with five other Bay Area counties. Santa Clara County has invested significant efforts in contact tracing, including, at its peak, over 900 contact tracers. In August 2020, ∼60 of these were bilingual Spanish-speaking contact tracers. Cases with language needs far outstripped the bilingual capacity of the CICT team (10). Due to the enormous time pressure to reach cases and sparsity of language information in laboratory reports, the assignment of cases to contact tracers did not account for patients’ language needs at baseline. Hence, the existing language skill set was dramatically underutilized. Instead, all contact tracers had the option of using a telephonic interpretation service provided by the state that connects callers with “qualified” interpreters, i.e., bilingual interpreters who have been tested and certified as proficient by a state agency or other testing authority (12). After reaching a patient and ascertaining interpretation needs, tracers would dial an interpreter into the call to conduct the interview with simultaneous interpretation. Numerous challenges surfaced in the process, including technical issues (e.g., dropped calls, poor audio quality, delays), longer holds and wait times for Spanish interpreters in particular, and questions about the mismatch between general interpreters and specific COVID-19 needs. These challenges disrupted the effectiveness of contact tracing with language minorities and the Spanish-speaking population in particular, which was the impetus for this particular project.

In this paper, we demonstrate how machine learning can leverage administrative data to create interpretable risk scores that an individual primarily speaks Spanish. The inputs from laboratory reports are age, name, and address. These inputs are merged with census data that contain Spanish-speaking information at the census block group (CBG) level, commercial data that contain language information with demographic correlates, and name-based race and ethnicity information from census and mortgage data. We show that an interpretable heuristic model can reasonably predict Spanish-speaking status, which was critical for adopting this approach in practice. These scores can then be used to identify patients who are in most need of a bilingual contact tracer, allowing for more efficient allocation of the relatively low supply of bilingual staff.

We evaluate the effects of utilizing this model in a randomized controlled trial of language matching with Santa Clara County’s actual contact-tracing process over a 2-mo period. During this period, CICT integrated our risk-scoring algorithm into their contact-tracing system and assigned patients with higher risk scores of being Spanish speakers to a language specialty team (LST) composed primarily of Spanish-speaking contact tracers. We tracked outcomes in real time and conducted a survey of the majority of CICT members on their experiences with language discordance. Using data recorded in the contact-tracing system and the survey responses, we assess the effects of language matching on interview times, ability to build rapport, patient satisfaction and trust, and effectiveness of interviews. Our work demonstrates substantial improvement from bilingual contact tracing compared to simultaneous telephonic interpretation.

Our contributions are threefold. First, we demonstrate how machine learning can be used with administrative data to improve health equity in contact tracing. Second, we evaluate the impact of a language-matching intervention in a randomized controlled trial and show that it enables improved and more equitable care. Third, our work illustrates the benefits of bilingual staff vs. interpretation services and the importance of quality of language access in leveling disparities in social services.

Based on the results and success of this trial, Santa Clara County has expanded language matching to all of CICT, and the state of California is contemplating adoption in the statewide system.

## Predicting Language Need from Sparse Records

### Features and Labels

We begin with laboratory reports of individuals who have tested positive for COVID-19. Due to a highly decentralized network of laboratories, the information that is consistently sent to Santa Clara County for each case is sparse, often comprising only the name of the patient, birth date, and residential address. Our aim is to use these inputs to develop a risk score of whether a patient is likely to prefer conducting the interview in Spanish. For expositional simplicity, we refer to such individuals as “Spanish speakers.”

We rely on US Census data to quantify the relationship between the likelihood of being a Spanish speaker and age, CBG, and last name. We also use a comprehensive list of first names derived from mortgage applications (13) to probabilistically infer race and ethnicity (14).

In addition, we use another source of administrative data to measure Spanish speaker status: voter registration data. Specifically, we use individual voter records of voters in Santa Clara County from L2, a private voter file vendor (15). These records include individual demographic information, including our features of interest, and language predictions from voter registration forms and a proprietary algorithm. Using these labels to create interpretable risk scores, we built an interpretable model that can be applied to all incoming cases, including nonregistered voters. For details, see *Materials and Methods*.

### An Interpretable Model of Language Need

While we implemented a fully machine-learned approach using random forests, interpretability was a priority for Santa Clara County and important for securing buy-in from Santa Clara County leadership and key stakeholders. A parsimonious set of features cohered with domain knowledge: For instance, Santa Clara County had previously attempted to assign cases from certain ZIP codes to teams with more language skills. We hence developed a data-driven heuristic approach using a small feature set that is transparent and understandable and yields improved predictive performance relative to the baseline.

**Fig. 1:**
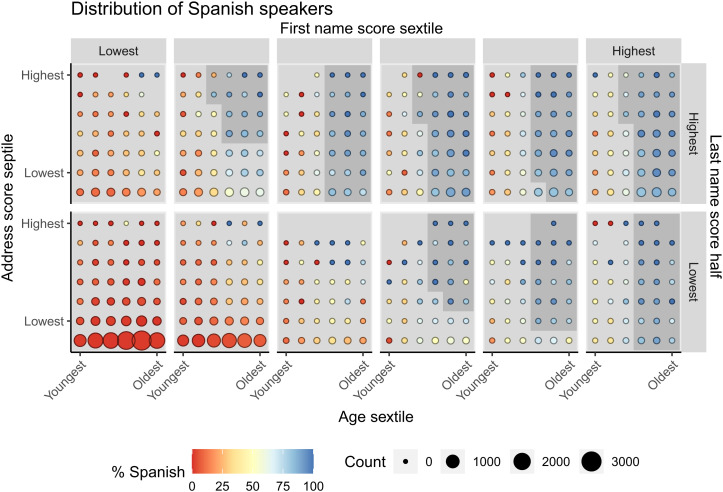
Distribution of Spanish speakers across all age, address score, first-name score, and last-name score bins in the train split of the L2 dataset. Each tile represents a bin that an individual may fall into based on the individual’s age, address score, first-name score, and last-name score. The size of the point in the bin corresponds to the total count of individuals in that bin. The color of the point corresponds to the percentage of individuals in that bin that are Spanish speakers. The gray shading represents the risk score cutoffs we use in our algorithm. Any individuals belonging to bins in the darker gray shade are flagged as Spanish speakers. Individuals in bins in the lighter gray shade are not flagged. A description of how the cutoffs are determined is in *SI Appendix*, *Methods*.

The heuristic approach optimally bins covariates based on predictive association with the Spanish label. Combinations of these bins are used to calculate risk scores (*Materials and Methods*). Fig. 1 visualizes the distribution of Spanish speakers across the four features. Within each panel, the *x* axis represents age from youngest to oldest, and the *y* axis represents the address score from lowest to highest. Across panels, Fig. 1 varies from lowest to highest the first-name score along the *x* axis and the last-name score along the *y* axis. Each dot represents the proportion of Spanish speakers in color, scaled by the number of individuals in that bin. We define this proportion as the risk score, i.e., the likelihood that an individual is a Spanish speaker. The modal patient, represented by the red dots in Fig. 1, *Bottom Left*, lives in an area with very few Spanish speakers with names not indicative of Latinx individuals. Fig. 1, *Top Right* indicates bins of individuals with a high likelihood of being Spanish speakers (blue colors). We define cutoffs based on operational capacity to select Spanish speakers for potential assignment to the treatment group. These are overlaid in dark gray shading in Fig. 1.

### Performance and Calibration

**Fig. 2:**
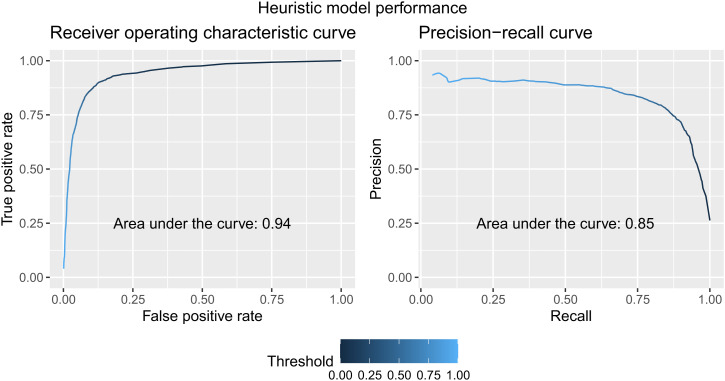
Performance of the heuristic model visualized as receiver operating characteristic and precision-recall curves along with AUCs. These curves are generated by evaluating the model at every classification threshold (depicted by the blue palette) on the test split from the L2 dataset. The high AUCs show the model’s ability to perform well in the context it was trained on.

We evaluate the performance of our heuristic algorithm in the test set of the voter data. Fig. 2 shows the receiver operating characteristic (ROC) and precision-recall (PR) curves for our algorithm at every classification threshold. With an area under the curve (AUC) of 0.94 for the ROC curve and an AUC of 0.85 for the PR curve, our algorithm performs well within the context it was trained on. At the threshold we selected (0.82) for operational purposes, the precision on the voter data test split is 88%.

## Evaluation Research Design

### Randomization

We assessed the impact of this intervention with a randomized controlled trial. We define the treatment of interest as whether a case is handled by the seven-member LST, composed primarily of contact tracers fluent in Spanish. The randomization scheme worked as follows: First, with each intake batch of new cases, we predicted which cases were likely to be Spanish speakers, based on whether the risk score exceeded a predetermined threshold set by operational capacity. Second, due to the limited capacity of the LST and the desire to allocate language skills where most needed, we randomized cases only in excess of a capacity threshold. If the number of flagged cases was below or equal to capacity at the time of data transfer, all of the cases were assigned to the LST. If the number of flagged cases was greater than capacity, we randomly assigned as many cases as the LST could handle and reserved the remainder of cases as the control group. Because case counts fluctuated dramatically during the study period, our analysis below accounts for this form of stratified randomization.

### Operation

Operationally, we embedded language matching within Santa Clara County’s existing case assignment process. CICT had already built data pipelines that allowed for the automated processing and routing of cases to different queues for specialty teams. For example, cases at long-term care facilities were identified and handled through a separate process. Cases were transferred in batches, with typically three batches per day, into the contact-tracing system. For our study, we added an additional language-matching step to this pipeline that took in unassigned cases, geocoded them to the CBG level, flagged cases as being likely Spanish speakers, and randomized and routed them to the LST in real time.

### Analysis

We examine outcomes of the contact-tracing process, including whether an interpreter was utilized, the timing of the process, whether an interview was completed, and the volume of information provided. If language matching had its intended effect, we should observe reductions in the reliance on interpreters, faster case processing, and improvement in case information collected (e.g., number of contacts provided).

The randomized design makes outcomes analysis relatively straightforward. We examine “intention-to-treat” (ITT) effects on outcomes of interest. The one complication is that the existing case assignment process broke down with the sharp case surge during the third wave of COVID-19 around December 2020. High case counts led to long transfer times from the statewide record system to Santa Clara County’s system, and Santa Clara County responded by assigning cases out immediately. This deviation meant that there was substantial “noncompliance” with the treatment assignment. We hence account for these deviations by using an instrumental variables (IV) approach to recover the “complier average causal effect (CACE),” namely the effect among the subgroup of cases that were routed to the LST solely because of the language-matching intervention. Since randomization occurred within each assignment batch, we account for this “block randomized design” throughout our analysis (16). Details on the approach and an alternate analysis using propensity score matching to account for the deviations are in *Materials and Methods*.

### Survey

To supplement the quantitative results, we administered a survey to all active contact tracers as of March 2021 (*n* = 645) about their experience with language discordance (see *SI Appendix*, Fig. S5 for questions). We received a total of 411 responses (64% response rate), which are summarized in *SI Appendix*, Fig. S6.

## Results

Our data consist of a total of 3,258 cases distributed across 64 assignment batches between 3 December 2020 and 6 February 2021. The average batch size was approximately *n* = 51. Of these 3,258 cases, 1,599 cases (49.08%) were routed to the LST. Because of the case surge associated with the third wave in December 2020, however, only 311 of these 1,599 cases (19.45%) were handled by the LST. Among the 1,659 cases that were assigned to the control group, 1,630 (98.25%) were handled by non-LST CICT staff.

While there was a primary LST group, if there were more cases assigned to the LST than they could handle (notwithstanding our capacity threshold in the randomization step), the LST team leads would occasionally assign those cases to non-Spanish speakers on related teams. We omit cases assigned to these “secondary” tracers from both the treatment and the control groups to isolate the effect of the primary LST. This reduces the sample of 3,258 cases down to 3,106 cases. (See *Materials and Methods* for results including those team members in the treatment group, with comparable results.)

### Covariate Balance

We assess covariate balance to verify whether randomization constructed comparable treatment and control groups in Table 1. For individual-level covariates, we assess balance on the Spanish risk scores and gender data from the contact-tracing system (CalCONNECT). We also merge an extensive set of socioeconomic covariates based on geocoded CBG from the 2019 American Community Survey 5-y estimates. Because CBG information based on address is required for language matching, we examine 3,055 cases with complete CBG-level information. We omit assignment batches with fewer than 2 cases in the treatment or control group, which results in 3,025 cases. We account for batch membership in assessing whether the estimated imbalance for each covariate is statistically significant.

**Table 1:** Balance on subset of covariates between cases randomly assigned to the LST and cases not selected between 3 December 2020 and 6 February 2021

	As randomized		
	Control	Treatment	*P* value
Individual-level variables			
Spanish propensity score	0.86	0.86	0.26
Male	0.53	0.52	0.31
CBG-level variables			
Social vulnerability index	74.55	75.44	0.41
Below poverty, %	7.42	7.54	0.76
Unemployed, %	3.51	3.49	0.41
Per capita income, $1,000	35.14	35.46	0.33
No high school diploma, %	24.93	24.66	0.55
Aged 65 y or older, %	11.35	11.32	0.39
Aged 17 y or younger, %	24.43	24.13	0.22
Civilian with a disability, %	11.09	11.05	0.97
Single-parent households, %	13.17	13.32	0.91
Minority, %	81.90	82.12	0.83
Sample size	1,601	1,424	

Shown is the balance check on a subset of individual-level and CBG-level variables to ensure that the treatment group (cases randomly assigned to the LST) and control group (cases not assigned to the LST) are comparable. The as randomized data contain units with nonmissing values for all balance covariates of interest. There are no units with missing data for the individual-level variables. There are 2 units with incomplete data for the CBG-level variables, and these are omitted. The sample used for the balance check consists of 64 batches.

We assess balance between treatment and control groups “as randomized.” Table 1 confirms that randomization worked as expected. Treatment and control groups are comparable on most dimensions. There are small imbalances on some CBG-level variables (e.g., residence in multiunit structures, vehicle possession), but such imbalances can be reasonably expected due to chance alone. See *SI Appendix*, Table S1 for the full set of covariates.

### Outcomes

As before, we begin by subsetting our sample of 3,025 cases with observed CBGs. Outcomes are defined based on fields present in CalCONNECT data for each case. We note at the outset that there are no discernible differences in missingness of outcomes between the assigned and control groups (*SI Appendix*, Table S3).

Table 2 presents causal effect estimates for the intention-to-treat effect in the left column and the CACE in the right column. We note two key results.

**Table 2:** Analyses on the effect of language matching on outcomes

	Intention-to-treat effect					Complier average causal effect		
Outcome	Control	LST	Effect	SE	*P* value	Effect	SE	*P* value
Interpretation service used	0.23	0.21	–0.03^* ^	0.02	0.07	–0.11	0.07	0.11
Interview completed	0.74	0.75	0.00	0.02	0.93	0.01	0.08	0.87
Refused to interview	0.02	0.01	–0.01^*^	0.00	0.05	–0.04^**^	0.02	<0.05
Refused to provide contacts	0.02	0.02	0.00	0.01	0.76	0.00	0.03	0.86
Time to interview completion, d	1.25	1.04	–0.14^***^	0.06	<0.01	–0.57 ^**^	0.27	0.04
Time case open, d	4.14	4.01	0.14	0.35	0.68	1.01	1.48	0.50
Interview completed within 24 h	0.65	0.68	0.04^**^	0.02	0.03	0.14	0.09	0.10
Interview completed on the same day	0.14	0.28	0.05^***^	0.01	<0.01	0.12 ^**^	0.06	0.03
Average number of contacts provided	0.19	0.20	0.03	0.04	0.44	0.08	0.15	0.60
At least one contact provided	0.08	0.08	0.00	0.01	0.74	0.00	0.04	0.94
Sample size	1601	1424						

*P < 0.10, **P < 0.05, ***P < 0.01. Shown is the effect of the intervention on cases assigned to the LST and cases not assigned to the LST (control). Batches with treatment or control groups smaller than two are dropped from the analyses. There were 62 batches in the ITT sample. For the ITT estimates, the average treatment effect (ATE) and SEs are estimated as shown in ref. 15. The CACE was estimated with the iv_robust() command in the estimatr package. First-stage results and diagnostic statistics are reported in *Materials and Methods*.

First, we find robust evidence from the IV analysis that the LST resulted in significant time savings. The time from a case being opened to the interview being completed is reduced by nearly 14 h, while the likelihood of the case interview being completed on the same day it is opened increased by 12%. These time savings are also consistent with the ITT analysis, which shows a 4% increase in the likelihood of the case interview being completed within 24 h of the time it is opened.

Second, we have moderate evidence that the LST reduces refusals to interview by 4% from the IV analysis and 1% from the ITT analysis, which is suggestive of improved patient engagement.

The ITT results also show a slightly significant decrease in usage of third-party interpreters of 3%. The IV analysis does not show a statistically significant decrease in usage of third-party interpreters, but the uncertainty interval is wide. We do not find evidence that language matching affected other quantitative measures of engagement, such as the number of contacts elicited or an affirmative refusal to provide close contacts.

## Survey Results

Survey responses corroborate the results above. First, while most respondents found the interpretation service valuable, respondents also noted common difficulties (e.g., dropped calls, lower rapport, and awkwardness with simultaneous interpretation).

Second, most respondents indicated that interpretation increased the time for contact tracing. Speed is critical for effective contact tracing, as there is a need to reach patients as quickly as possible after a diagnosis, and 45% of respondents indicated that the time added was “considerabl[e]” or “a great deal.”

Third, two-thirds (67%) of bilingual respondents reported that conducting the interview themselves was much easier (54%) or somewhat easier (13%). Respondents also reported several common benefits from being able to use their own non-English language skills, including 1) increased client satisfaction; 2) improved ability to control the interview, elicit relevant information, and provide other assistance (e.g., referrals to supportive services); 3) ability to build trust, comfort, and openness; and 4) reduced interview times.

## Discussion

Our study has provided robust evidence that machine learning can improve the efficiency and effectiveness of pandemic response by matching cases to contact tracers based on language. While much concern has been articulated about how machine learning can encode bias, this use case powerfully illustrates how machine learning can be deployed to promote public health equity.

We note several limitations of the study. First, while the research design was rigorous, it was not possible to anticipate the extreme strain that the third wave of COVID-19 imposed on the contact-tracing process. This is what accounts for degrees of noncompliance with the original protocol. Nonetheless, the results strongly suggest material benefits to language matching.

Second, because some LST members were drawn from the base of contact tracers, our estimates may somewhat inflate the benefits of language matching. After all, the LST members, who represent about 10% of all Spanish-speaking tracers, would have been able to engage Spanish-speaking cases in the absence of the intervention. However, the impetus for this trial was precisely the observation that language skill sets were heavily underutilized, as cases were not systematically matched in any way. In the control group, roughly 12% of cases would have encountered a Spanish speaker by chance alone, so our intervention demonstrates that language matching can effectively utilize existing language capacity.

Third, numerous refinements could be made to the language algorithm. Most importantly, there may be benefits from tuning the model more closely to the patient population based on contact-tracing interviews. One challenge to doing so, however, is that contact tracing occurs under substantial time pressure, such that language fields are not always consistently recorded. This is why our early monitoring of whether the algorithm in fact flagged cases with Spanish-speaking needs was important in implementing the approach.

Fourth, this intervention can be seen as an interim measure until Santa Clara County is able to routinely and clearly route language data collected at the point of intervention (i.e., testing sites) to the electronic data collection systems. This work and the challenges encountered also demonstrate the need for data systems that are built to allow for timely analysis of demographic data so that even in situations of high demand, existing language skills can be fully utilized.

Fifth, this intervention could be improved by accounting for fluidity in the language and communication preferences of Spanish speakers when collecting language data. The language preferences of an individual may not capture those of the individual’s household, especially if there are multigenerational family members involved, and individuals may prefer a different language for discussing health concepts than the one they report when being asked for their language preference or their fluency in English (17). Training staff to record language information that reflects a patient’s preferences in the health setting rather than a rigid assessment of the patient’s English proficiency would make it more feasible to provide language-concordant care and also allow for a more refined language algorithm in this context.

We conclude by noting the real promise of this intervention, which was implemented under the severe circumstances of a pandemic, with staggering growth in cases and considerable operational constraints. COVID-19 has had a dramatically disparate impact across different demographic groups. We have shown that this light-touch intervention—utilizing an interpretable machine-learning approach—leveled language differences at a critical point in pandemic response.

## Materials and Methods

### Details on Language-Matching Algorithm

The language-matching algorithm was used for all results reported in the main text. The algorithm uses census and mortgage data to quantify the relationship between the inputs and the likelihood of Spanish-speaking status, and it is trained and validated on voter registration data. Then, we use an algorithm to select cutoffs for determining which risk scores were eligible for being routed to the LST, which allowed us to target an estimated caseload for the LST while still optimizing for precision. We also compare the algorithm’s performance against a fully machine-learned random forest approach using the same features and training and validation data. More details on the implementation and random forest’s performance can be found in *SI Appendix*, *Methods*.

### Deployment

Additional work was done to deploy the language-matching algorithm in the field and embed it in Santa Clara County’s existing contact-tracing pipeline. First, the algorithm expects CBG as an input to calculate a risk score for a given individual, so offline geocoding was performed via record linkage to a list of addresses in Santa Clara County to acquire CBG data for any case record addresses.

Second, the algorithm needed to be integrated with Santa Clara County’s contact-tracing pipeline, which used the CalCONNECT database. As a part of our partnership with Santa Clara County, we reviewed the pipeline and made improvements to speed up the processing of new cases, which was necessary for the deployment of the algorithm to effectively route cases to the LST.

Third, we closely monitored the language-matching algorithm’s performance and solicited feedback from all contact tracers using it when we first deployed it in the Santa Clara County system. This was to check that the algorithm was actually effectively flagging Spanish speakers in the field.

Details on all of the aforementioned topics can be found in *SI Appendix*, *Methods*.

### Propensity Score Matching

To account for the deviations in how contact-tracing interviews were conducted during the third wave of COVID-19 and the lower adherence with the assignment protocol in that time period, we conducted an additional matched analysis using propensity score techniques to construct a balanced matched sample (18). In this approach, the propensity score for each case was modeled with a logistic regression using the risk score and a subset of the CBG-level covariates used in the balance assessment. Details on the matched analysis and the results can be found in *SI Appendix*, *Methods*.

### Missingness

To ensure that we were not selecting outcomes that were heavily imbalanced between the treatment and control groups, we compared the proportion of missingness for each outcome variable in *SI Appendix*, Table S3. Based on this analysis there were no statistically significant imbalances.

### Sensitivity to LST Membership

Because cases assigned to the LST were not always routed to team members due to unavailability or overflow, they were sometimes routed to tracers on the periphery of the team. These individuals were aware of the study and the LST’s purpose and may have been part of the team for short periods, but were not primary members of the LST. In *Results*, we focus on the program effect of being routed to the LST by omitting assigned cases routed to these secondary team members from the treatment and control groups. As a sensitivity check we perform the same analyses but keep cases routed to these secondary team members.

We assess covariate balance on this new dataset in *SI Appendix*, Table S4. As before, there are a few small imbalances on some CBG-level variables, but this can be reasonably expected due to chance alone.

*SI Appendix*, Table S5 shows the three types of analyses performed: as randomized, matched, and IV. In the as randomized analysis, the significant effects on decreases in interviewing time for cases remain. The matched analysis retains the decrease in interpretation service usage and increase in likelihood to complete the interview, and the IV analysis retains the effects showing patients being less likely to refuse interviews and interviews being more likely to be done sooner. Most of the results hold at less pronounced levels, which would be expected given the nature of individuals not on the LST.

### IV Analysis

Due to deviations from the assignment protocol during the case surge associated with the third wave, our research design can be understood as an experiment with two-sided noncompliance (19). In the IV analysis, we include batch fixed effects and several preassignment covariates in the two-stage least-squares estimation. The required identification assumptions for estimating the CACE are noninterference, random assignment, excludability, relevance, and monotonicity (19). It is unlikely that there was meaningful interaction between cases that participated in the pilot, since the CICT process handled each case in isolation, and random assignment to the LST (*Z*) is satisfied by design. Major violations of the exclusion restriction also seem unlikely. Patients did not have prior awareness or explicit knowledge of their participation in the pilot or their assignment to the LST. With respect to relevance, the first-stage results and diagnostics demonstrate that *Z* has a strong, nonzero effect on whether the case was actually handled by LST staff (*D*). The *F* statistic is 315.73, with a *P* value of less than 2.2e-16. Finally, in assessing the plausibility of the monotonicity assumption, the observed frequency of noncompliance in the control group is instructive. 1,288 of the 1,599 (80.55%) of the cases assigned to the LST (*Z_i_* = 1) were handled by non-LST CICT staff (*D_i_* = 0). Only 29 of the 1,659 (1.75%) cases assigned to the control group (*Z* = 0) were handled by the LST (*D* = 1). Given the way in which cases were routed during the third wave, it is difficult to imagine that a meaningfully large group of cases would have systematically defied their assignment status *Z*.

## Data Availability

Anonymized R object (RDS) and comma-separated values (CSV) files with postprocessed census data and generated risk scores from the paper and R code for all algorithms described and analysis carried out in the paper have been deposited in Harvard Dataverse (DOI: 10.7910/DVN/SF606L). Some study data are available. Voter data from L2 (https://l2-data.com/), provided through an agreement with Stanford Libraries (https://searchworks.stanford.edu/view/12357569), may be accessible for the purposes of validation or peer-review if Stanford Libraries is first contacted to seek permissions for these purposes. Data from the Santa Clara County Public Health Department cannot be shared as they contain individual-level protected health information.
